# Reducing ACL injury risk: A meta‐analysis of prevention programme effectiveness

**DOI:** 10.1002/ksa.12542

**Published:** 2024-11-12

**Authors:** Clemens Clar, Stefan F. Fischerauer, Andreas Leithner, Laura Rasic, Paul Ruckenstuhl, Patrick Sadoghi

**Affiliations:** ^1^ Department of Orthopaedics and Trauma Medical University of Graz Graz Austria

**Keywords:** anterior cruciate ligament, knee injury, prevention programme

## Abstract

**Purpose:**

The aim of this study was to conduct a meta‐analysis of the literature regarding anterior cruciate ligament (ACL) injury prevention programmes (IPPs) in order to assess the effectiveness of ACL prevention programmes based on current high‐quality studies. The hypothesis was that the implementation of ACL IPPs significantly reduces the incidence of ACL ruptures compared to standard practice.

**Methods:**

A meta‐analysis of the literature was conducted using the databases PubMed, EMBASE, MEDLINE, CINHAL and Cochrane Central Register of Controlled Trials. The search terms utilized were ACL, injury, knee, control and prevention. The collected data and reported clinical outcomes were independently gathered by three different individuals. After evaluating the heterogeneity of the studies, the DerSimonian–Laird random effects models were employed to determine the pooled risk ratios (RRs) and the risk differences (RDs) regarding ACL Injuries. The RD was utilized to ascertain the number needed to treat.

**Results:**

The search strategy identified 743 studies, of which 11 met all inclusion and quality criteria for pooled analysis. The total number of study participants was 16,316. The overall RR of sustaining an ACL injury in the intervention group was 0.36 (95% confidence interval [CI]: 0.23 to 0.57) of the control group, showing a significant reduction in the ACL injury risk of the intervention group (*p* < 0.001). We identified an RD of −1.4% (95% CI: −2.4% to −0.4%) in favour of the intervention group. The number needed to treat in preventing one ACL rupture was 71.

**Conclusion:**

In conclusion, the study clearly demonstrates a significant positive preventive effect of training programmes concerning ACL injuries (*p* < 0.001). The pooled estimates indicate that such programmes result in a significant reduction of ACL injury risks (*p* < 0.001). Despite the moderate quality of the included literature, the results exhibit robustness. However, based on the literature examined, no definitive superior training programme could be identified.

**Level of Evidence:**

Level II.

**Abbreviations**
ACL: anterior cruciate ligament
CCTR: Cochrane Controlled Trial Register
CI: confidence interval
IPP: injury prevention programme
LE: lower extremity
NNH: number needed to harm
PEP Programme: Prevent Injury and Enhance Performance Programme
RD: risk difference
RR: risk ratio

## INTRODUCTION

Anterior cruciate ligament (ACL) injuries are among the most common sports injuries today, resulting in over 2 billion dollars in healthcare costs annually in the United States [2, 3]. Due to the doubling of these injuries over the past 20 years, research in sports medicine has increasingly focused on ACL injury prevention programmes (IPPs). For prevention programmes to be effective, both intrinsic and extrinsic risk factors must be considered during planning, alongside physiological, biomechanical and socioeconomic factors. The current trend in ACL IPPs is moving towards dynamic loading and strengthening through proprioceptive or neuromuscular training [2, 9, 15, 16, 20, 35].

Even though cruciate ligament reconstruction is considered state‐of‐the‐art, the discourse between operative and non‐operative treatment of ACL ruptures is on‐going, notably because both treatment approaches have undergone a continuous process of refinement and optimization over the years [17, 22, 28, 29, 31, 32]. Some studies indicate that immediate ACL reconstruction and subsequent rehabilitation did not yield better outcomes than primary conservative therapy with optional ACL reconstruction at a later stage [10, 17, 29].

About 70%–80% of ACL ruptures are attributable to minimal or non‐contact injuries, with the most common motion mechanism involving a slightly flexed knee joint in valgus position during pivoting, jumping and cutting [4, 5, 7, 8]. This type of injury warrants attention, as a significant percentage of these injuries can be effectively mitigated through appropriate preventive measures and targeted multifaceted training programmes. These programmes focus on high‐risk populations, such as elite athletes or young female athletes, with the objective of enhancing risky movement patterns, like the landing phase after jumping and improving neuromuscular feedback [15, 21, 26, 30].

The aim of this study is to conduct a meta‐analysis of the current literature on prevention programmes for ACL ruptures in both male and female athletes. Through a meta‐analysis, the intention was to examine the extent to which these programmes lower the risk of sustaining an ACL rupture. The hypothesis was that the implementation of ACL IPPs significantly reduces the incidence of ACL ruptures compared to standard practice.

## MATERIALS AND METHODS

### Systematic research and strategy

To assess our hypothesis, we executed a meta‐analysis on controlled trials, amalgamating data to evaluate the efficacy of ACL IPPs. Employing a systematic approach, we reviewed the literature through online databases including PubMed, MEDLINE, EMBASE, CINAHL (Cumulative Index of Nursing and Allied Health) and CCTR (Cochrane Controlled Trial Register). Our electronic search entailed specific keywords such as ‘anterior cruciate ligament OR knee OR injury AND control OR prevention AND injury’ as in the study conducted in 2012 [33]. This study was pre‐registered at PROSPERO (Registration number: CRD42024590683).

We exclusively incorporated prospective, controlled trials that directly investigated the efficiency of an ACL IPP by comparing the results with a control group in human beings. In instances of data overlap, efforts were made to merge studies to the extent possible, we excluded fully overlapping studies. Additional exclusions comprised duplicate studies, those not centred on clinical outcome or treatment, animal studies, studies lacking any intervention, and those with unacceptably high attrition rates (>20%). Eligible interventions encompassed proprioceptive techniques (such as PEP, Prevent Injury and Enhance Performance Programmes) and neuromuscular facilitation training, with or without the inclusion of a balance board, wobble board or round board.

### Extraction of relevant data

The eligibility of studies was independently and doubly assessed by three independent individuals (SF, CC and PS) with crosschecking to mitigate errors. Any disagreements were resolved through discussions or, if requisite, with the involvement of the senior author. The bibliography of every included study was thoroughly examined for additional relevant research. Data extraction from the included studies involved study design, patient characteristics, and the endpoint incidence of ACL rupture, with the process carried out in duplicate by two individuals, followed by crosschecking. All search activities were conducted in September 2023.

### Assessment of quality

To assess the quality of the individual studies, the ROBINS‐I tool was used. Among the 11 included studies, the following seven domains were categorized as ‘low risk’, ‘moderate risk’, ‘serious risk’ or ‘critical’: bias due to confounding, bias in the selection of participants into the study, bias in the classification of the intervention, bias due to deviations from intended interventions, bias due to missing outcome data, bias in the measurement of the outcome, and bias in the selection of the reported result [34].

#### Study heterogeneity

Quantification of heterogeneity was conducted by using the *I* ^2^ index [14]. The existence of between‐study heterogeneity was evaluated using Cochrane's *Q* homogeneity test, with a *p* value of 0.10 employed to address the limited statistical power of this test in smaller sample sizes [36, 37].

### Quantitative data synthesis

For data aggregation, we formulated random‐effects models utilizing the DerSimonian–Laird random effects method [7]. These models propose that the observed heterogeneity among studies in a meta‐analysis arises from normally distributed individual effects around a common impact. This assumption was visually examined in forest plots. The data were combined to generate pooled risk differences (RDs) and pooled risk ratios (RRs). The RD was utilized to calculate the number needed to treat (NNT = 1/RD), representing the number of patients that must be enroled in the prevention programme to prevent one ACL tear. The number needed to harm (NNH) is provided for studies indicating a better outcome without any prevention.

All calculations were executed by using Intercooled STATA® 17.0 BE (StataCorp LP). The significance level for pooled estimates was set at *α* = 5%.

## RESULTS

### Study characteristics

**Figure 1:**
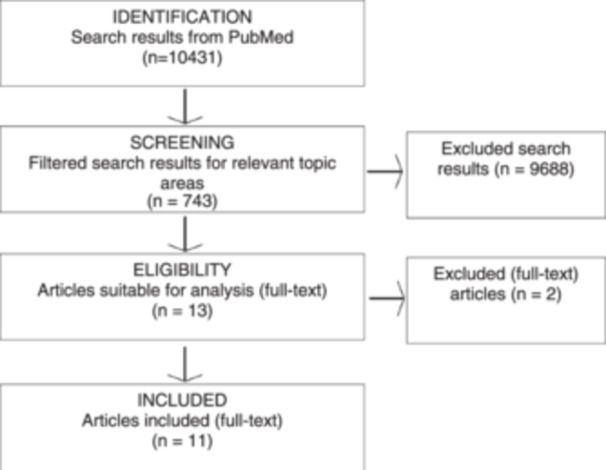
The flowchart illustrates the search results based on the search string. After identifying the relevant studies, they were filtered according to relevant topics. Subsequently, the studies were checked for their suitability for analysis, with all criteria needing to be met before a total of 11 studies could be included.

Through specific literature research, 743 studies were identified. After filtering out duplicates, studies without a focus on clinical outcomes or treatment, studies involving non‐human species, or studies without a specific intervention, 11 studies remained for analysis [1, 6, 11, 12, 13, 18, 19, 24, 25, 27, 38] (Figure 1). The publication period of these 11 studies is reported to be between 1996 and 2018, and they were published in both German and English languages. Four studies exclusively investigated football players, three focused on handball players, two studies examined athletes in general, and one study each explored football and basketball players or football, volleyball and basketball players collectively. Consequently, the studies were analysed based on the examined gender, with seven studies exclusively involving women, two studies involving men, and two studies including both genders.

### Description of the included studies

See Table 1.

**Table 1:** The 11 included studies were analysed based on the following points: Study name and year of publication, study design, level of evidence (GRADE), participants, intervention group, control group, time period, type of intervention, frequency of training and treatment effect.

Study name and year of publication	Study design	Level of evidence (GRADE)	Participants	Intervention group	Control group	Time period	Types of intervention	Frequency of training	Treatment effect
Achenbach et al. (2018)	RCT	Moderate	279	Injury‐prevention programme (neuromuscular exercises)	No intervention	One season	Neuromuscular exercises	2–3×/week during preseason and 15 min 1×/week during competition period	Incidence of severe knee injury (0.04/1000 h vs. 0.33/1000 h)
Waldén et al. (2012)	Stratified cluster RCT	Moderate	4564	Neuromuscular warm‐up programme (15 min, core stability, balance, knee alignment)	No intervention	7 months	Neuromuscular warm‐up	Twice a week	Anterior cruciate ligament (ACL) injury rate (0.28% vs. 0.67%)
LaBella et al. (2011)	Cluster RCT	Low	1492	Neuromuscular warm‐up (20 min, coach‐led)	Usual warm‐up	One season	Neuromuscular warm‐up	80.4% compliance (1425/1773 practices)	Lower extremity injury rates (0.43 vs. 1.22 per 1000 AEs)
Gilchrist et al. (2008)	Cluster RCT	Low	1435	Neuromuscular warm‐up	No intervention	One season	Neuromuscular control warm‐up	3 times per week	ACL injury rate (0.057 vs. 0.189 per 1000 AEs, 70% reduction)
Pfeiffer et al. (2006)	Prospective cohort study	Low	1439	Plyometric‐based exercise (focus on landing mechanics)	No intervention	Two consecutive seasons	Plyometric‐based exercise programme	Twice a week	Noncontact ACL injury rate (0.167 vs. 0.078 per 1000 exposures)
Petersen et al. (2005)	Prospective controlled study	Low	276	Prevention programme (injury mechanism info, balance‐board, jump training)	Usual training	One season	Proprioceptive and neuromuscular training	Regular (weekly)	ACL injury rate (0.04 vs. 0.21 per 1000 h)
Mandelbaum et al. (2005)	Prospective cohort study	Moderate	2946 (year 1); 2757 (Year 2)	Sports‐specific training (education, stretching, strengthening, plyometrics, agility)	Traditional warm‐up	Two years	Neuromuscular and proprioceptive training	Regular during training	ACL injury reduction (88% in 2000, 74% in 2001)
Petersen et al. (2002)	Prospective controlled study	Low	Two teams 2nd division	Proprioceptive and neuromuscular training (injury mechanism info, proprioception, jump training)	No intervention	One season	Proprioceptive and neuromuscular training	Preseason + continued during season	No severe ankle or knee injuries; reduced light and medium injuries
Heidt et al. (2000)	Prospective cohort study	Low	300	Preseason conditioning programme (7 weeks)	No intervention	One year	Conditioning programme	7 weeks pre‐season	Lower incidence of injuries; 2.4% ACL injuries in trained vs 3.1% in untrained
Hewett et al. (1999)	Prospective cohort study	Low	1263	Plyometric training programme	No intervention	6 weeks	Plyometric training	3 days/week	Knee injury incidence (0.12 per 1000 AE in trained vs 0.43 in untrained)
Caraffa et al. (1996)	Prospective cohort study	Moderate	600	Proprioceptive training (5 phases of increasing difficulty)	No intervention	Three seasons	Proprioceptive training (wobble‐boards)	20 min per day	ACL injury incidence (1.15 per team per year)

Abbreviation: RCT, randomized controlled trial.

### Publication bias

**Figure 2:**
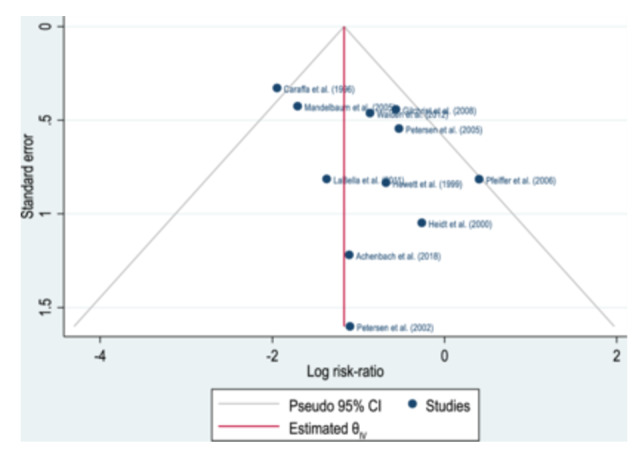
Funnel plot depicting the 11 included studies. This figure graphically illustrates publication bias by plotting the standard error (SE) on the *x*‐axis and the logarithm of the risk ratios (RRs) on the *y*‐axis against the RR. The blue dots represent the respective studies, the vertical red line represents the pooled RR, and the grey diagonal line shows the corresponding 95% confidence interval. Although the studies are somewhat skewed, all except the study by Caraffa et al. fall within the funnel, suggesting low publication bias. Furthermore, this was mathematically calculated as not evident (*p* = 0.094).

The funnel plot (Figure 2) reveals a discernible bias, attributed partly to the exclusion of a few available studies, which could not meet all the inclusion criteria mentioned above, and partly to the study by Pfeiffer et al. [27]. However, the potential for publication bias was quantitatively assessed using Egger regression, yielding no significant evidence of publication bias in the included studies (*p* = 0.094).

### Heterogeneity

**Figure 3:**
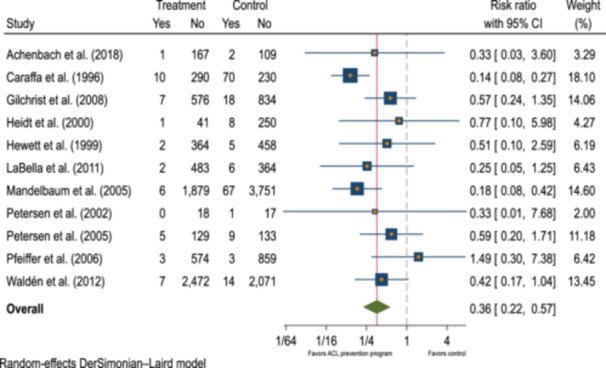
Forest plot illustrating the findings of the meta‐analysis. The included 11 studies were listed in a table and analysed in terms of treatment versus control group, risk ratio (RR) with 95% confidence interval (CI), and percentage weight. The size of the blue squares represents the percentage weight of each respective study, and the length of the horizontal line of each study represents the respective RR with 95% CI. The green diamond on the bottom represents the overall effect with 95% CI.

The between‐study variation of the effect sizes is evident from the forest plot (Figure 3). The heterogeneity statistic *I* ^2^ was about 37% (95% confidence interval [CI]: 0.0%–70%), corresponding to a small to medium variability of the effect size that was caused by between‐study differences. The homogeneity test of study‐specific effect sizes was also rejected with a Cochran's *Q* test statistic of 16 and a *p* value of 0.11 and did not reveal a statistical in‐between study heterogeneity.

### Overall effect

The overall mean effect‐size estimate under the DerSimonian–Laird method was 0.36 with a 95% CI from 0.23 to 0.57) for the 16,316 examined athletes across 11 studies. We found this effect to be significant with *p* < 0.001, indicating a preventive effect of specific training programmes on ACL injury risk. The variance shared between studies was 0.20. The study by Caraffa et al. [6] exhibited the lowest overall effect at 0.14 (95% CI: 0.08–0.27) and showed the highest weighting of 18%. Conversely, the study by Pfeiffer et al. [27] displayed the highest value at 1.5 (95% CI: 0.30–7.4).

### Risk difference

In the overall RD, studies also demonstrated a preventive effect, with the highest value observed in the study by Caraffa et al. [6] at −0.20 (95% CI: −0.25 to −0.15). Only the study by Pfeiffer et al. [27] exhibited a non‐negative overall RD, at 0.002 (95% CI: −0.005 to 0.009). The average of all examined studies stands at −0.014 (95% CI: −0.024 to −0.004). The calculated number needed to treat to prevent one ACL injury was therefore 71.

#### Study quality

**Figure 4:**
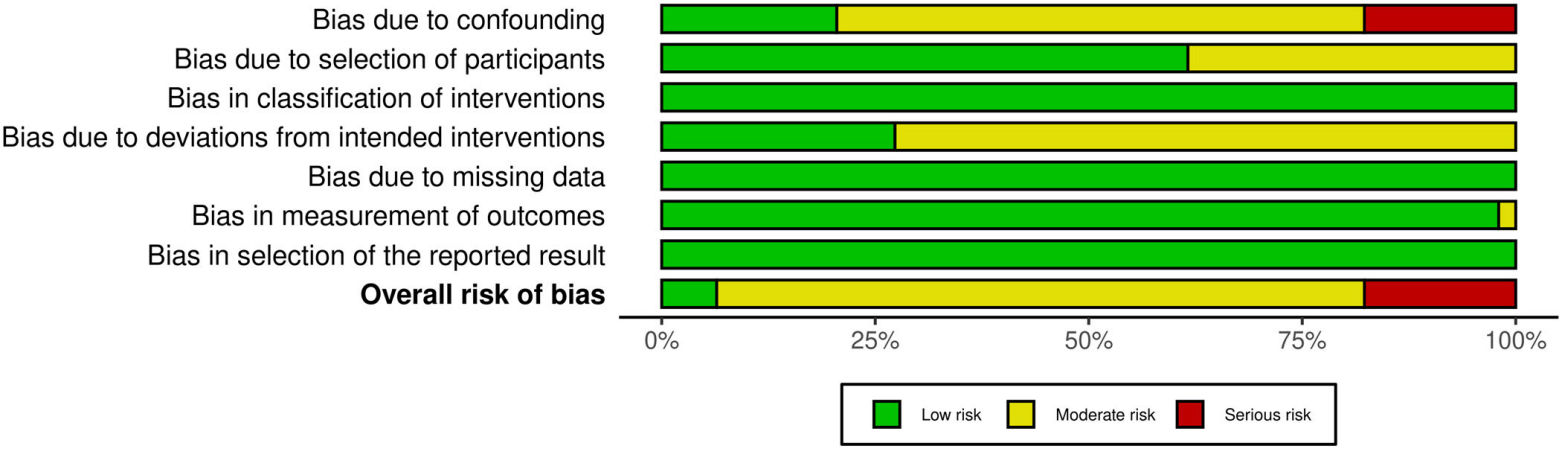
The percentage distribution of the ‘low risk’, ‘moderate risk’ and ‘serious risk’ ratings among the 11 included studies by using the ROBINS‐I tool. The listed seven domains were analysed individually for each study, and subsequently, the overall risk was determined.

The overall assessment using the ROBINS‐I tool for the 11 included studies showed that one study (LaBella et al. [18]) had a ‘low risk’ of bias. Eight studies (Achenbach et al. [1], Gilchrist et al. [11], Pfeiffer et al. [27], Petersen et al. [24, 25], Mandelbaum et al. [19], Hewett et al. [13] and Caraffa et al. [6]) demonstrated a ‘moderate risk’, while two studies (Waldén et al. [38] and Heidt et al. [12]) were found to have a ‘serious risk’ of bias (Figure 4, Table 2).

**Table 2:** Risk of bias assessments (ROBINS‐I tool).

Study	D1	D2	D3	D4	D5	D6	D7	Overall	Weight
Achenbach et al. (2018)	Moderate	Low	Low	Moderate	Low	Low	Low	Moderate	3.29
Caraffa et al. (1996)	Moderate	Low	Low	Moderate	Low	Low	Low	Moderate	18.10
Gilchrist et al. (2008)	Low	Low	Low	Moderate	Low	Low	Low	Moderate	14.06
Heidt et al. (2000)	Serious	Low	Low	Low	Low	Low	Low	Serious	4.27
Hewett et al. (1999)	Moderate	Moderate	Low	Moderate	Low	Low	Low	Moderate	6.19
LaBella et al. (2011)	Low	Low	Low	Low	Low	Low	Low	Low	6.43
Mandelbaum et al. (2005)	Moderate	Moderate	Low	Low	Low	Low	Low	Moderate	14.60
Petersen et al. (2002)	Moderate	Low	Low	Low	Low	Moderate	Low	Moderate	2.00
Petersen et al. (2005)	Moderate	Moderate	Low	Moderate	Low	Low	Low	Moderate	11.18
Pfeiffer et al. (2006)	Moderate	Moderate	Low	Moderate	Low	Low	Low	Moderate	6.42
Waldén et al. (2012)	Serious	Low	Low	Moderate	Low	Low	Low	Serious	13.45

*Note*: Seven bias domains, D1–D7, were analysed for each study and the overall bias risk was determined.

Abbreviations: D1, Bias due to confounding; D2, Bias due to selection of participants; D3, Bias in classification of interventions; D4, Bias due to deviations from intended interventions; D5, Bias due to missing data; D6, Bias in measurement of outcomes; D7, Bias in selection of the reported result; ROBINS‐I, Risk Of Bias In Non‐randomized Studies—of Interventions.

## DISCUSSION

The study's hypothesis is considered to be the most important finding of this meta‐analysis, demonstrating a significant preventive effect of ACL IPPs, with an overall effect size of 0.36 and an RD of −0.014, indicating that such programmes effectively reduce the risk of ACL injuries. Heterogeneity was moderate, but no significant publication bias was detected. The number needed to treat to prevent one ACL injury was 71, highlighting the practical impact of these programmes.

### Summary of evidence

Upon analysis of the included manuscripts, a significant disparity in conclusions among the authors was evident: Caraffa et al. [6], Hewett et al. [13], Heidt et al. [12], Petersen et al. [24, 25], LaBella et al. [18], Waldén et al. [38] and Achenbach et al. [1]. were able to demonstrate a significant positive effect through various prevention programmes in their intervention groups. Mandelbaum et al. [19] and Gilchrist et al. [11] concluded that there was only a benefit in terms of injury reduction from the investigated prevention programmes, but they did not provide explicit recommendations. In contrast, Pfeiffer et al. [27] concluded that a twice‐weekly 20‐min plyometric‐based training showed no significant advantage in preventing ACL injuries among female high‐school athletes in non‐contact sports. However, the authors did not elucidate why these results present a significant contrast to the otherwise published literature.

Pooling the individual‐included studies in this meta‐analysis reveals that preventive training programmes have significant and unequivocal evidence for a positive impact on ACL injuries. Furthermore, the analysed pooled RR was 0.36 (95% CI: 0.23–0.57), indicating a significant reduction in the ACL injury risk of the intervention group. It should be noted that the study by Caraffa et al. [6], which analysed all ACL injuries of the subjects in contrast to the others focusing on either non‐contact or contact injuries, may introduce some bias. However, our analysis found no significant deviation in the reported outcome of these two studies compared to the others analysed.

As early as 1996, Caraffa et al. [6] demonstrated that the risk of an ACL tear could be mitigated through specific ACL training programmes. Since then, research on this topic, including numerous meta‐analyses, has consistently increased. The findings of this study, particularly regarding the significant effect of these programmes, are further supported by recent comparative studies. For instance, Al Attar et al. [2] conducted a meta‐analysis that evidenced a 71% reduction in risk attributable to plyometric exercises. Similarly, Naderi et al. [23] revealed through a comparable meta‐analysis that handball players without an ACL prevention programme face a 66% higher risk of ACL tears. Another comparative study by Huang et al. [15] yielded analogous results, indicating that ACL IPPs generally reduce the risk of an ACL tear by 53%.

In general, the scientific quality of the studies can be considered as low. However, compared to the meta‐analysis conducted in 2012, the quality has been improved. Only 2 of the total 11 studies, namely Pfeiffer et al. [27] and Petersen et al. [24, 25], were able to indicate sufficient blinding of the studies. In contrast, five studies demonstrated adequate randomization (Gilchrist et al. [11], Heidt et al. [12], LaBella et al. [18], Waldén et al. [38] and Achenbach et al. [1]). Matched pair analysis was conducted by Caraffa et al. [6] and Mandelbaum et al. [19].

### Limitations

For the present study, several limitations and shortcomings need to be addressed. As with all meta‐analyses, it is important to acknowledge that the quality and interpretability heavily depend on the primary literature, and therefore the validity is strongly linked to this literature. As with all meta‐analyses, inclusion and exclusion criteria are not identical in the included manuscripts. In order to illustrate potential discrepancies, inclusion and exclusion criteria in a table describing the individual studies were added. Although the scientific quality of the included studies is considered relatively low, it should be noted that such quality is not uncommon for studies in the field of musculoskeletal and surgical trials [33]. The study by Mandelbaum et al. [19] was included in the analysis of the work despite the high attrition rate, as it is a milestone with respect to the Fifa11+ programme and therefore requested by the majority of readers. Heterogeneity assessment was performed to compare groups according to the main endpoints, without calculating for additional subgroups. This was done in line with the previous work, published 10 years ago [33] to compare changes of the findings.

Next, the heterogeneity of training programmes in the primary included studies should be mentioned, which necessitated the use of random‐effects models, a legitimate approach in such works. Due to this heterogeneity, we are not able to describe specific factors of an optimal training programme. Furthermore, it is worth noting that 8 of the 11 included studies examined neuromuscular training, seven explicitly focused on women, and five studies included the sport of football, which could introduce some bias. However, such bias should have been evident in the forest plot through a distinct cluster. On the contrary, our forest plot displayed a uniform distribution of the included studies around the pooled RR [33].

## CONCLUSION

In conclusion, the study clearly demonstrates a significant positive preventive effect of training programmes concerning ACL injuries, as the pooled estimates indicate that such programmes significantly reduce the risk of ACL injury. Despite the moderate quality of the included literature, the results exhibit robustness. However, based on the literature examined, no definitive superior training programme could be identified.

## AUTHOR CONTRIBUTIONS

**Clemens Clar:** Conceptualization; methodology; analysis of data; investigation; data curation; writing—original draft preparation; preparation of revision. **Stefan F. Fischerauer:** Conceptualization; methodology; analysis of data; investigation; data curation; supervision; writing—review and editing. **Andreas Leithner:** Conceptualization; methodology; supervision; writing—review and editing **Laura Rasic:** Conceptualization; methodology; analysis of data; investigation; data curation. **Paul Ruckenstuhl:** Conceptualization; methodology; supervision, writing—review and editing **Patrick Sadoghi:** Conceptualization; methodology; analysis of data; investigation; data curation; supervision; writing—review and editing. All authors have read and agreed to the published version of this manuscript.

## CONFLICT OF INTEREST STATEMENT

The authors declare no conflict of interest.

## ETHICS STATEMENT

This meta‐analysis was conducted in accordance with ethical guidelines and standards established for research synthesis in the medical field. As a secondary analysis of previously published data, this study did not involve direct contact with human participants or require the collection of new primary data. Therefore, institutional review board (IRB) approval and informed consent from patients were not applicable.

## Data Availability

Additional data from this study is available upon request from the corresponding author or the first author.
